# Modulation of the Epithelial Sodium Channel (ENaC) by Bacterial Metalloproteases and Protease Inhibitors

**DOI:** 10.1371/journal.pone.0100313

**Published:** 2014-06-25

**Authors:** Michael B. Butterworth, Liang Zhang, Xiaoning Liu, Robert M. Shanks, Patrick H. Thibodeau

**Affiliations:** 1 Department of Cell Biology, University of Pittsburgh, School of Medicine, Pittsburgh, Pennsylvania, United States of America; 2 Department of Ophthalmology, University of Pittsburgh, School of Medicine, Pittsburgh, Pennsylvania, United States of America; University of Giessen Lung Center, Germany

## Abstract

The serralysin family of metalloproteases is associated with the virulence of multiple gram-negative human pathogens, including *Pseudomonas aeruginosa* and *Serratia marcescens*. The serralysin proteases share highly conserved catalytic domains and show evolutionary similarity to the mammalian matrix metalloproteases. Our previous studies demonstrated that alkaline protease (AP) from *Pseudomonas aeruginosa* is capable of activating the epithelial sodium channel (ENaC), leading to an increase in sodium absorption in airway epithelia. The serralysin proteases are often co-expressed with endogenous, intracellular or periplasmic inhibitors, which putatively protect the bacterium from unwanted or unregulated protease activities. To evaluate the potential use of these small protein inhibitors in regulating the serralysin induced activation of ENaC, proteases from *Pseudomonas aeruginosa* and *Serratia marcescens* were purified for characterization along with a high affinity inhibitor from *Pseudomonas*. Both proteases showed activity against *in vitro* substrates and could be blocked by near stoichiometric concentrations of the inhibitor. In addition, both proteases were capable of activating ENaC when added to the apical surfaces of multiple epithelial cells with similar slow activation kinetics. The high-affinity periplasmic inhibitor from *Pseudomonas* effectively blocked this activation. These data suggest that multiple metalloproteases are capable of activating ENaC. Further, the endogenous, periplasmic bacterial inhibitors may be useful for modulating the downstream effects of the serralysin virulence factors under physiological conditions.

## Introduction

Opportunistic pathogens secrete multiple virulence factors to modulate interactions with the host, to acquire nutrients from the environment and to facilitate adhesion and colonization to a variety of substrates [1]–[4]. *Pseudomonas aeruginosa* secretes a series of proteases that target host proteins to modulate the immune response and to facilitate colonization in infected tissues. Bacterial adherence and colonization may be facilitated by the degradation of host immune and signaling proteins that would otherwise initiate or potentiate the host response. Alternatively, remodeling the local environment of a bacterium may promote its adherence or growth.

Alkaline protease (AP) has been shown to play a role in multiple modes of *Pseudomonas* infection [2], [4]–[8]. The AP is a member of the serralysin family of proteases, belonging to the larger M10 family of Zn^2+^ metalloproteases [9], [10]. Structurally, AP is composed of two globular domains: an N-terminal catalytic domain and a C-terminal Ca^2+^-binding domain. The N-terminal catalytic domain contains the canonical HEXXHXXGXXH motif associated with Zn^2+^ coordination in the metalloproteases. This domain shows significant structural similarity to a variety of metalloproteases, including the human matrix metalloproteases. The C-terminal domain of AP contains multiple Ca^2+^-binding motifs associated with the Repeats-in-ToXin (RTX) family of bacterial virulence factors [11]–[13]. This domain has previously been shown to bind Ca^2+^, which induces its folding and the folding and activation of the N-terminal protease domains [14]–[16]. Calcium is tightly coordinated by conserved asparatate- and glycine-rich nonapeptide RTX repeats, as seen in the high-resolution structures of *Pseudomonas* AP and serralysin (SmP) from *Serratia marcescens* [9], [10]. This mode of Ca^2+^-mediated regulation is likely a key feature of the serralysin proteases, as well as the family of RTX virulence factors and toxins [11], [12], [17].

Bacterial species producing serralysins often co-express inhibitors that putatively block the function of their respective proteases in the cytosol or periplasm [18], [19]. In *Pseudomonas aeruginosa*, the *apr* operon is genomically encoded proximal to a high-affinity inhibitor, *aprI*, of the protease gene, *aprA* [20]. This inhibitor (AP Inh) is localized to the periplasm of the bacterial cells and putatively protects the cells from the AP enzymatic activity. It is unclear how this occurs as the protease is not normally localized to the periplasm [21]. Structural studies have shown that this inhibitor binds in the active site of AP and is coordinated by the Zn^2+^ ion and other protein-protein contacts with the surfaces of the N-terminal catalytic domain [19], [21]. Further, these studies suggest that the inhibitor binds with high affinity (4 pM) to the AP active site [19].

The active AP has previously been correlated with the virulence of *Pseudomonas* in cystic fibrosis (CF) [4], [7], [8], [22], [23]. Similarly, expression of the serralysins from *Serratia marcescens* has been shown to exacerbate corneal injury [24], [25]. While the pathophysiological mechanisms in patients have not been fully elucidated, AP has been shown to cleave bacterial flagellin, host signaling molecules and the epithelial sodium channel (ENaC) [26]–[28]. Cleavage of flagellin and cytokines would putatively alter the host response to the pathogen, while ENaC cleavage would be predicted to remodel the airway surface hydration state, reduce muco-cilliary clearance, and facilitate bacterial adherence and colonization. The combined effects of blunting the host immune response and altering ion channel activity would putatively contribute to an increase in bacterial load within the airway and the apparent virulence of the pathogen.

To evaluate the potential use of the *aprI* inhibitor as a modulator of AP activity in airway epithelial cells, AP and AP Inh were purified. Tight association and protease inhibition were measured *in vitro* and demonstrated that near stoichiometric addition of the inhibitor completely bound the protease and inhibited its activity. This inhibition was blocked with N-terminal fusions to the inhibitor, consistent with the known structures of the protease-inhibitor complexes [18], [19]. ENaC-mediated sodium transport in a model cell line and primary airway cultures confirmed that AP addition to the apical bathing surface activated ENaC and that near stoichiometric addition of AP Inh blocked the observed ENaC activation. Similarly, ENaC activation was observed in response to apical addition of serralysin from *S. marcescens*. This activation was blocked by the addition of the purified AP Inh protein. These data show that multiple M10/serralysin family members can activate ENaC and more broadly implicate the M10 protease family as modulators of ENaC activity. Further, the native *Pseudomonas* inhibitor is effective as an inhibitor for multiple M10 proteases under physiological conditions. These results suggest that serralysin-mediated ENaC activation requires active protease and that modulation of these protease activities could potentially be leveraged to effectively reduce the virulence associated with bacterial metalloprotease production and secretion.

## Materials and Methods

### Protein expression and purification

Alkaline protease from *Pseudomonas aeruginosa* was purified under denaturing conditions and refolded as previously described using a T7 regulated pET vector for expression [16]. Serralysin from *Serratia marcescens* (SmP) was similarly expressed and purified under denaturing conditions using a pBAD expression vector with arabinose induction. Briefly, donor cultures were grown overnight at 37°C under antibiotic selection. The donor cultures were used to inoculate 1 liter expression cultures that were grown to mid-log phase (OD_600_∼0.6–0.8) before induction. Induction was accomplished with the addition of 1 mM IPTG or 0.02% w/v arabinose. Proteins were expressed at 37°C for 4–6 hours and cultures were harvested by centrifugation. Cells were resuspended (50 mM Tris, 150 mM NaCl, pH 7.2) lysed by sonication and inclusion bodies were collected by centrifugation (14, 000 G RCF, 30 minutes, 4°C). The insoluble material was resuspended (50 mM Tris, 150 mM NaCl, 6 M GuHCl, pH 7.2) and loaded on a Ni-NTA column (GE Life Sciences) equilibrated in the resuspension buffer. The column was washed with resuspension buffer supplemented with 60 mM imidazole and the protein was eluted with 400 mM imidazole in resuspension buffer. The denatured protein was further purified using a sephacryl S200 Hi-Prep gel filtration column (GE Life Sciences) (50 mM Tris, 150 mM NaCl, 6 M GuHCl, pH 7.2) and stored at 4°C before refolding and use.

The *aprI* inhibitor (Inh) was PCR amplified from PAO1 genomic DNA (ATCC) and cloned into a pET vector as a C-terminal fusion to a 6xHis Smt3 tag [29]. The protein was expressed as described above for AP and was purified from the supernatant after lysis (50 mM Tris, 150 mM NaCl, pH 7.2) and centrifugation at 40,000 G RCF for 30 minutes at 4°C. The protein was loaded onto a Ni-NTA column equilibrated in lysis buffer. The column was washed in buffer supplemented with 60 mM imidazole and eluted in buffer supplemented with 400 mM imidazole. The Smt3 fusion was cleaved using Ulp1 protease at 4°C and the resulting products were separated using a sephacryl S200 Hi-Prep column and a second Ni-NTA column. The N-terminal Smt3 and Ulp1 protease were immobilized on the second Ni-NTA column while the AP Inh inhibitor passed through the column without binding. The Smt3 N-terminal tag was left intact as a fusion to AP Inh for experiments evaluating the specificity of the inhibitor binding to AP (Inh*).

### Protease refolding, activity and inhibition assays

The AP and SmP proteases were refolded as previously described [16], [26]. Briefly, proteases in GuHCl were rapidly diluted into refolding buffer (50 mM Tris, 150 mM NaCl, 2 mM CaCl_2_) on ice for 15 minutes. The refolding reactions were clarified by centrifugation (21,000 G RCF, 15 minutes, 4°C) or by filtration (100 kDa MWCO, Ultracell concentrators, Millipore) prior to use. Protease activity was assessed using fluorescently labeled protease substrates. A highly conjugated casein protein (EnzCheck, Invitrogen) and a metalloprotease specific peptide substrate (Mca-K-P-L-G-L-Dpa-A-R-NH2; Mca: (7-methoxycoumarin-4-yl)acetyl, Dpa: N-3-(2, 4-Dinitrophenyl)-L-2,3-diaminopropionyl; R&D Systems) were chosen based on previous studies of AP. Protease activities were assessed, in the presence and absence of AP Inh, in 96 well plate format using a BioTek Synergy 4 multi-mode plate reader in endpoint or kinetic mode and velocity parameters were fit using Gen5 software (BioTek). Analytical gel filtration experiments were performed using a TOSOH QC-PAK GFC 200 column on a Shimadzu Prominence HPLC. The column was pre-equilibrated using the refolding buffer in the presence or absence of Ca^2+^ before the protein injection. Protein elution was monitored by UV absorbance at 220 and 280 nm.

### Cell culture and electrophysiology

Fisher rat thyroid (FRT) cells (ATCC) were maintained in DMEM/F12 media supplemented with 5% FBS, 10 mU/ml TSH, 0.01 mg/ml insulin, 10 nM hydrocortisone, 0.005 mg/ml transferrin, 10 ng/ml somatostatin, 10 ng/ml glycyl-L-histidyl-L-lysine acetate. FRT Cultured mouse cortical collecting duct (mCCD) kidney epithelial cells were kindly provided by Bernard Rossier and Laurent Schild, Université de Lausanne, Switzerland, and maintained as previously described [30]. Dr. Joseph Pilewski of the University of Pittsburgh Cystic Fibrosis Research Center Cell Core provided primary human bronchial epithelial (HBE) cells, under a protocol approved by The University of Pittsburgh Investigational Review Board [26], [31]. Primary cells were routinely maintained as previously described. For short-circuit current (*I_SC_*) recordings, cells were grown on Transwell inserts until a confluent, high–resistance monolayer was obtained. These inserts were mounted in modified Ussing chambers (P2300, Physiological Instruments) in a modified Ringer's solution and continuously short-circuited with an automatic voltage clamp (VCC MC8, Physiological Instruments) as previously described [32]–[34]. Transepithelial resistance was monitored throughout the recording by periodically applying a 2 mV pulse via an automated pulse generator. Recordings were digitized and analyzed using PowerLab (AD Instruments, Colorado Springs, CO).

To isolate the action of the serralysin proteases from serine proteases, which are known to activate ENaC, cells were preincubated with 10 µM camostat mesylate (Santa Cruz Biotechnology) for 60 minutes prior to recordings. Camostat was maintained in the apical hemi-chamber (10 µM) during serralysin activation and did not inhibit the activity of the AP and SmP proteases when compared to cells without camostat addition (not shown) [35]. Prior to trypsin addition, the apical chamber was washed with a five-fold volume exchange of warmed Ringer's solution to remove the camostat. The surface ENaC that was uncleaved was subsequently activated by exogenous trypsin addition (1 µM) as we have demonstrated [26]. No trypsin activation was observed in recordings where the camostat was not removed from the apical chamber, confirming the inhibition of a serine protease under the experimental conditions.

A typical *I_SC_* recording included a 10-minute equilibration period, followed by stimulation with trypsin, AP or serralysin, with and without AP Inh. *I_Na_* was determined by the addition of 10 µM amiloride to the apical cell chamber at the end of each recording. Maximal ENaC activity was generated by trypsin addition and recorded *I_SC_* was normalized to the maximum trypsin-activated *I_Na_*. Protease concentrations were chosen to match the apparent specific activities using the fluorescent assays described above and as previously described.

### Cell surface cleavage, biotinylation and western blotting

Cleavage of γ-ENaC at the apical surface was accomplished using a modified double-immunoprecipitation protocol. FRT cells were grown on 75 cm^2^ flasks (Thermo Fisher) to obtain sufficient cells to seed ∼3×10^6^ cells per filter. Cells were harvested and transfected in suspension using Lipofectamine 2000 (Life Technologies) following manufacturer's protocols. Cells were incubated with the lipid/DNA complex for 5 hours before seeding directly onto 6-well filter supports at superconfluency. A total of 4 transwell filters (Corning 6-well, 2.4 cm diameter) were used for each condition and pooled following biotinylation to obtain a single sample. For the required 4 filters (1.2×10^7^ cells) equal concentrations of untagged α- and β-ENaC and tagged γ-ENaC were used at a total DNA quantity of 10 µg [36]. Medium was replaced after 6 hours to fully supplemented medium (including FBS) as described previously [33]. The FRT cells were allowed to form a polarized monolayer, as confirmed by transepithelial resistance, before surface biotinylation labeling [31].

To cleave surface ENaC, cells were treated with alkaline protease as described for electrophysiological experiments. As a control, trypsin was added to the apical surface. FRT cells were incubated with protease and/or inhibitor for 30 minutes. Inserts were then washed five times with ice-cold PBS containing Mg^2+^ and Ca^2+^ (PBS+CM). The cells were biotinylated at 4°C in borate buffer (85 mM NaCl, 4 mM KCl, 15 mM Na_2_B_4_O_7_, 375 µg biotin, pH 9) on the apical surface with the basolateral side of the monolayer bathed in medium containing 10% FBS to prevent basolateral biotinylation. After 20 min, cells were aspirated and PBS+CM containing 10% FBS was placed on the cells to quench the biotinylation. Monolayers were washed five times with ice-cold PBS+CM with agitation, and the cells were lysed in 400 µl biotinylation lysis buffer (0.4% sodium deoxycholate, 1% NP-40, 10 mM Tris-base, 50 mM EGTA, protease inhibitor cocktail II (Roche)) per filter. Post-nuclear supernatant was obtained for immunoprecipitation (IP) of ENaC using an anti-V5 antibody (Novus Biologicals) conjugated to 100 µl protein-G beads. Samples were incubated with rotation at 4°C overnight. Following IP beads were washed three times in PBS containing 1% triton-X and 0.01% SDS. The isolated ENaC IP samples were next eluted from the beads by incubating in 120 µl biotinylation lysis buffer containing 30 µl 10% SDS for 10 min at room temperature. The eluate was incubated with 75 µl pre-washed streptavidin magnetic beads (Pierce/Thermo Scientific) at 4°C overnight with rotation to enrich for the cell surface, biotinylated ENaC. Samples were washed three times in biotinylation lysis buffer during magnetic bead separation. After the final wash, beads were resuspened in buffer with 30 µl of a 2 X sample buffer including 100 mM DTT heated to 95°C for 8 min and separated on a 8–16% Tris-HEPES-SDS-PAGE precast gel (Bio-Rad). Samples were transferred to nitrocellulose membranes (Millipore) using a Trans-Blot SD Semi-Dry Transfer Cell (Bio-Rad) following the manufacturer's protocols and assessed by western blotting. Western blots were quantified after digital capture (scanning) using Adobe Photoshop CS.

### Statistics and curve fitting

All data were analyzed using SigmaPlot (Systat, Chicago, IL). Differences in *in vitro* protease activities were assessed using Welch's analysis of variance or student's t-test. Differences in summarized electrophysiological data were evaluated by t-tests with p<0.05 considered statistically significant. Activation curves were fit using SigmaPlot, and rate constants derived from the plotted exponential rise curves (R-squared values ranged from 0.85 to 0.99).

## Results

### ENaC cleavage by active alkaline protease

**Figure 1:**
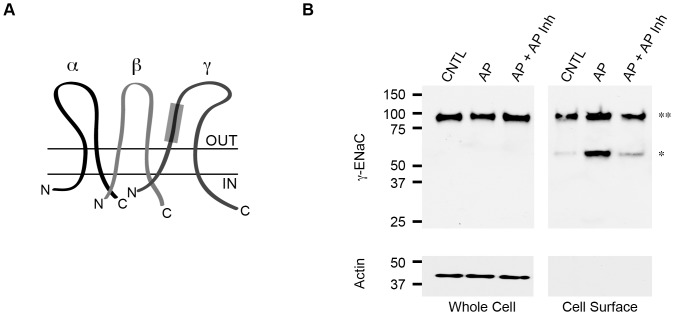
ENaC cleavage by active AP. Cell surface cleavage of γ-ENaC was assessed biochemically using purified AP and AP Inh. ***A***, a cartoon schematic of the ENaC channel is shown with the α-, β-, and γ-subunits labeled. The protease sensitive tract in γ-ENaC is shown as a gray box and spans residues 139 to 181 [49]. ***B***, cell surface cleavage of ENaC was assessed by western blotting. Total ENaC is shown for control cells (CNTL), AP treated cells, or cell co-treated with AP and AP Inh from whole cell lysates, *Whole Cell*. The cell surface ENaC was assessed after biotinylation and streptavidin capture, *Cell Surface*. From the cell surface pool of ENaC the full length (**) and cleaved (*) forms of the channel are observed. Actin was used as a loading control and was not observed in biotinylated samples.

The functional ENaC channel exists in the plasma membrane as a putative trimer, normally composed of α-, β- and γ-subunits (Figure 1A). Trafficking and recycling of ENaC at the plasma membrane regulate channel function. In addition, proteolytic cleavage of sequences within the α- and γ-subunits has been shown to functionally activate the channel in the plasma membrane. Our previous studies of ENaC activation demonstrate that the *Pseudomonas aeruginosa* Zn^2+^ metalloprotease, alkaline protease, is capable of activating ENaC in multiple primary and model epithelial cells [26]. This activation required the presence of the γ-subunit and its protease-sensitive inhibitory peptide, as mutant γ-ENaC constructs lacking this regulatory sequence failed to show a response to treatment with purified AP. The α-ENaC protease sites did not appear to be functionally sensitive to the addition of AP in Ussing chamber experiments.

To assess the cleavage state of γ-ENaC in response to treatment with the active AP, ENaC was expressed in confluent monolayers of FRT cells. FRT cells were chosen as they robustly express ENaC and can be transfected to provide sufficient channel protein for cell surface analysis. Following transfection, polarization and expression, purified AP was added to the apical cell surface and allowed to cleave plasma membrane-resident protein. The cells were then biotinylated and ENaC was immunoprecipitated. Cell surface ENaC was captured by streptavidin pull-down and the eluate was subsequently assessed by western blotting.

Under basal conditions, ENaC expressed robustly in FRT cells (Figure 1B, *Whole Cell*). Isolation of the cell surface ENaC protein allowed for the identification of ENaC proteolysis following treatment with either AP or AP+AP Inh. Control cells, which were left untreated with protease or treated solely with protease inhibitors, showed two distinct ENaC bands. A high molecular weight band, corresponding to full-length γ-ENaC, was identified at ∼90 kDa (Figure 1B, Cell Surface, **). A second and less intense band was observed at ∼55 kDa and corresponded to a small population of ENaC that was cleaved at the cell surface under control conditions (Figure 1B, Cell Surface, *). This band likely arose from cleavage events in the biosynthetic pathway or plasma membrane that were not fully inhibited with the camostat pretreatment.

The purified AP and AP Inh proteins were then assessed for their ability to regulate γ-ENaC cleavage biochemically. The addition of AP to the apical surface of the FRT cells resulted in a dramatic increase in the amount of cleaved γ-ENaC observed by western blotting. This increased cleavage resulted in a strong enhancement of the ∼55 kDa band seen by western blotting. Densitometric analyses of the cleaved bands showed 54%±10% of the cell surface ENaC to be cleaved under these conditions. This was consistent with the previously reported proteolytic activation of γ-ENaC. To assess the potential use of the *Pseudomonas* AP inhibitor, both the AP and AP Inh were co-incubated on the FRT cells. The inclusion of the AP Inh protein at 2∶1 stoichiometry to the concentration of AP resulted in a decrease in observed ENaC cleavage, as seen by western blotting. Densitometric analyses of the cleaved ENaC demonstrated that 23%±6% of the cell surface ENaC protein was cleaved (p<0.05 compared to AP treatment). These data demonstrated that the AP Inh protein effectively inhibited AP activity outside of the bacterial cell and could be used to block ENaC cleavage.

### Calcium-regulated folding and activation of the serralysin proteases

**Figure 2:**
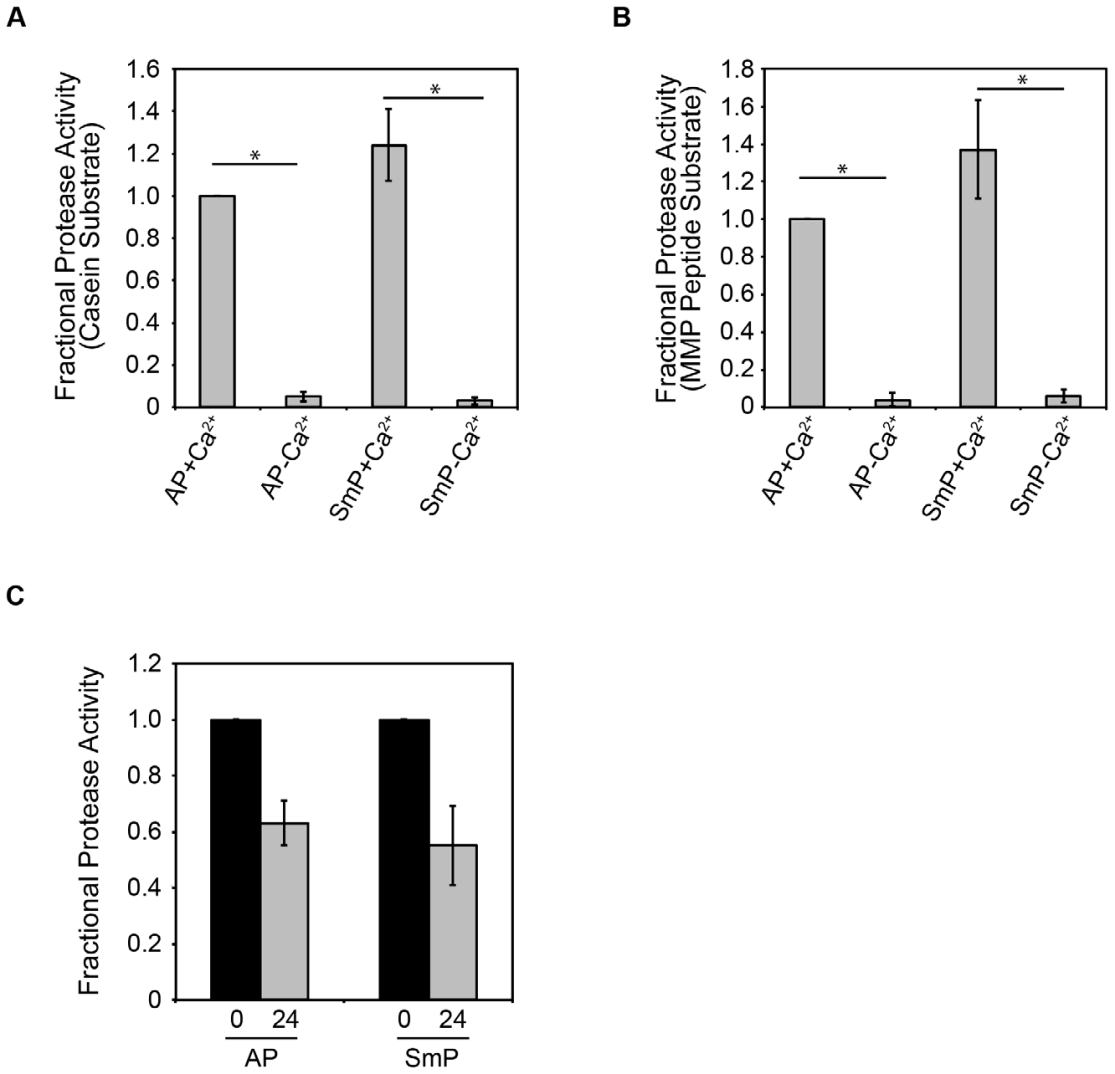
Folding and activation of serralysins from *Pseudmonas aeruginosa* and *Serratia marcescens*. Protease activities were assessed for both AP and SmP using a fluorescently-conjugated casein substrate, ***A***, or a fluorescent metalloprotease peptide substrate, ***B***. Addition of 2 mM Ca^2+^ to the refolding buffers resulted in robust folding and activation of both AP and SmP, as measured by protease activity. ***C***, protease activity was assessed immediately after refolding or following a 24 hour incubation at 37°C using the metalloprotease peptide substrate. Buffer controls are subtracted from all samples and the activities are normalized to AP, ***A, B***, or protease activity measured immediately after refolding, ***C***. Data shown are mean ± standard deviation from n = 6 experiments. P<0.001, *.

To evaluate the potential that other bacterial metalloproteases in the serralysin family could activate ENaC and be regulated by the *Pseudomonas* protease inhibitor, proteases from *Pseudomonas aeruginosa* and *Serratia marcescens* were purified for *in vitro* characterization and electrophysiological studies. Previous studies of the folding of AP demonstrated that Ca^2+^-binding within the C-terminal RTX repeats regulated protease folding with an apparent affinity of 50–60 µM [16]. To evaluate the Ca^2+^ induced folding of the serralysin proteases, AP and SmP were purified and refolded *in vitro*. Previous studies have shown that this procedure is efficient, with >85% of the protein being refolded in the presence of saturating Ca^2+^ [16]. The function of both refolded proteases was assessed using fluorescence-based assays to confirm activity. Two assays using different substrates were chosen utilizing either fluorophore-conjugated casein or a fluorophore-conjugated peptide. In the presence of Ca^2+^, both AP and SmP showed significant protease activities when compared with samples refolded without Ca^2+^ (p<0.001) (Figure 2A). At equimolar concentrations, SmP showed a modest increase in activity relative to AP using the casein substrate. The SmP apparent activity was 123%+/−14% when compared to the activity of the refolded AP. In the absence of Ca^2+^, both proteases showed minimal activity against the casein substrate, consistent with the previously described Ca^2+^-regulated folding and activation of the RTX proteases.

Similarly, both proteases showed a Ca^2+^-dependent activity using a peptide substrate (Figure 2B). The peptide substrate was chosen based on the structural similarity between AP, SmP and mammalian matrix metalloproteases (MMP2 and MMP9) and putatively provides a substrate with fewer structural constraints. Millimolar concentrations of Ca^2+^ resulted in the activation of both AP and SmP as detected by kinetic and endpoint assays. As with the casein substrate, the SmP activity was consistently higher than that of AP with the fluorogenic peptide. Using the peptide substrate, the SmP produced 138%+/−24% that activity of the refolded AP. In the absence of Ca^2+^, only trace amounts of protease activity could be detected (p<0.001). The casein and peptide proteolysis data demonstrate that both the AP and SmP are regulated by Ca^2+^ binding and can be similarly refolded *in vitro*.

To evaluate the stability of the refolded proteases, the proteins were incubated for extended periods at 37°C and proteolytic activity was subsequently evaluated. Similar auto-proteolysis/protease competition experiments have been previously utilized to probe the stabilities of other bacterial exoproteins [37]. Proteolytic cleavage of the enzyme leads to a decrease in activity, which is related to the accessibility of cleavage sites and the stability of the native state. Once folded, both the AP and SmP proteases showed sustained activities after incubation at physiological temperatures (Figure 2C). Following 24 hours incubation at 37°C, AP retained 63%+/−8% activity. Similarly, SmP retained 54%+/−14% activity after a 24-hour incubation. The long-lived activity demonstrated that secretion of these enzymes would provide for sustained catalytic activity outside of the cell.

### Binding and inhibition of AP by its inhibitor from *Pseudomonas*

The RTX proteases are often co-expressed with endogenous inhibitors that putatively protect the bacterial cell from unwanted protease activity [18], [19]. Previous studies have shown that the inhibitor from *Pseudomonas aeruginosa* binds with high affinity to AP. To evaluate the potential use of the inhibitor outside of the bacterial cell, the inhibitor was purified for *in vitro* binding studies and physiological experiments on epithelial cells.

Two forms of the inhibitor were produced with differing N-termini. In *Pseudomonas*, the inhibitor is secreted into the periplasm using an N-terminal signal sequence. The N-terminal residue, generated after cleavage of the signal sequence in the periplasm, is coordinated in the protease active site by the Zn^2+^ cofactor [19]. The inhibitor was expressed as a Smt3-fusion in *E. coli* with the purification tag fused to the N-terminus in lieu of the signal sequence. This fusion (AP Inh*) would putatively block binding of the inhibitor to the active site of the protease and served as a control for the inhibitor studies. Removal of the Smt3 provided the N-terminus required for binding to the AP active site and provided an active form of the inhibitor (AP Inh).

**Figure 3:**
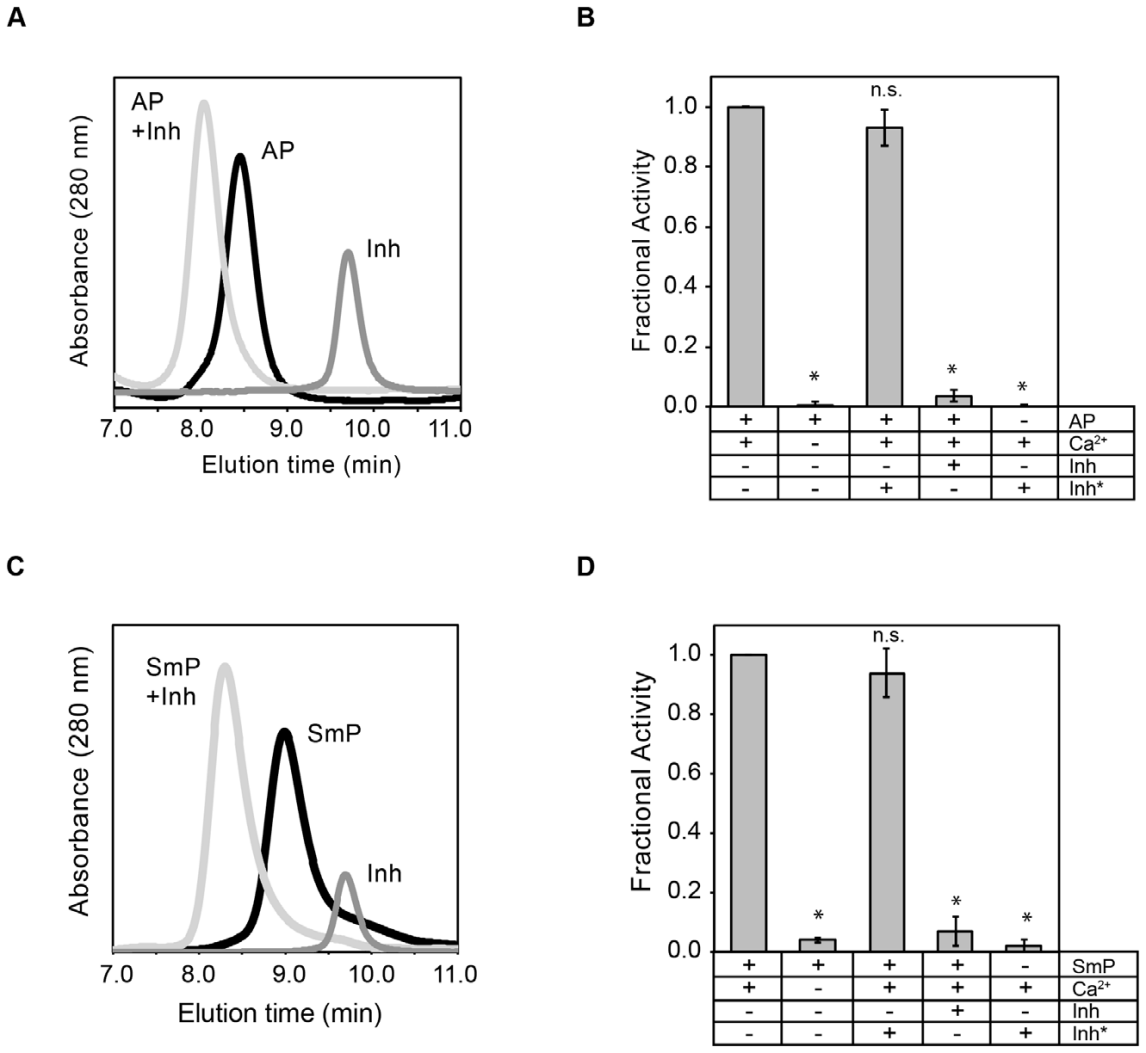
Binding and inhibition of AP Inh to AP and SmP. Inhibition of protease activity was evaluated using purified AP and the *Pseudomonas aeruginosa* AprI inhibitor. ***A***, analytical gel filtration was used to assess complex formation between the refolded AP and the purified inhibitor. Representative chromatograms are shown for protease, *black line*, the inhibitor, *dark gray line*, and the complex, *light gray line*. **B**, activity assays were performed using the fluorescent peptide substrate for AP. ***C***, analytical gel filtration was used to assess complex formation between the refolded SmP and the purified inhibitor. Representative chromatograms are shown for the protease, *black line*, the inhibitor, *dark gray line*, and the complex, *light gray line*. **D**, activity assays were performed using the fluorescent peptide substrate for AP. Data shown are mean ± standard deviation for n = 5 experiments. P<0.001, *; n.s., not significant.

Binding of the inhibitor to AP and SmP was assessed using analytical gel filtration and activity assays. The refolded proteases ran as single symmetric peaks, as monitored by UV absorbance of the column eluate (Figure 3A, C). Similarly, the inhibitor eluted as a single, symmetrical peak when injected alone onto the column. When the inhibitor was mixed in equimolar ratios with the protease, the individual inhibitor and protease peaks decreased to baseline and a new species appeared at a lower retention time. Binding appeared to be tight as the peak shift appeared to be complete and the shift resulted in a near symmetrical single peak that represented protease bound to the inhibitor.

To assess the activities of AP and SmP in the presence of the inhibitor, the fluorescence-based peptide activity assay was utilized (Figure 3B, D). Activation of both AP and SmP was dependent on Ca^2+^, as shown in Figure 1. The inclusion of Inh* showed minimal effect on the activity of the AP or SmP. Small decreases in apparent protease activity may be associated with a non-fluorescent protein being included in the reaction and a dilution of the reporter substrate. In contrast, inclusion of the inhibitor at equimolar concentrations significantly reduced protease activity by greater than 90% for both AP and SmP (p<0.001). This was consistent with the binding seen in the analytical gel filtration. The inhibitor alone, either Inh or Inh*, showed no protease activity. Extended incubation (12–16 hours at 37°C) of both proteases with the inhibitor did not lead to an increase in protease activity, suggesting that inhibitor binding was tight and that the co-incubation did not result in a decrease in the effectiveness of the inhibitor over the time courses evaluated (data not shown).

### Functional Regulation of ENaC by AP, SmP and the serralysin inhibitor in mCCD cells

Previous studies of AP indicated that the protease, when expressed by *Pseudomonas* or purified from recombinant sources, was capable of activating ENaC [26]. To assess the effects of both SmP and inhibitor, mCCD cells were chosen for electrophysiological studies. These cells were chosen as they express ENaC at high levels, form tight monolayers, and act as a robust model for physiological measurement of ENaC activity [30]. Further, we have previously shown that AP robustly activates ENaC in this cell line. Cells were pretreated with camostat mesilate, an inhibitor of serine proteases, to isolate the activities of AP and SmP on the cell surface [35]. This inhibition results in an accumulation of ENaC channels at the cell surface that can be evaluated by addition of exogenous proteases [38]. The proteases, either alone or in combination with the AP inhibitor were added to the apical baths of Ussing chambers. As shown previously, AP was capable of activating ENaC when *I_SC_* was measured in Ussing chambers. Trypsin (1 µM) was used as a control to provide maximal ENaC activation (complete cleavage) for each experiment. Amiloride addition to the apical bath inhibited the protease-sensitive currents, consistent with the measured current being mediated by ENaC.

**Figure 4:**
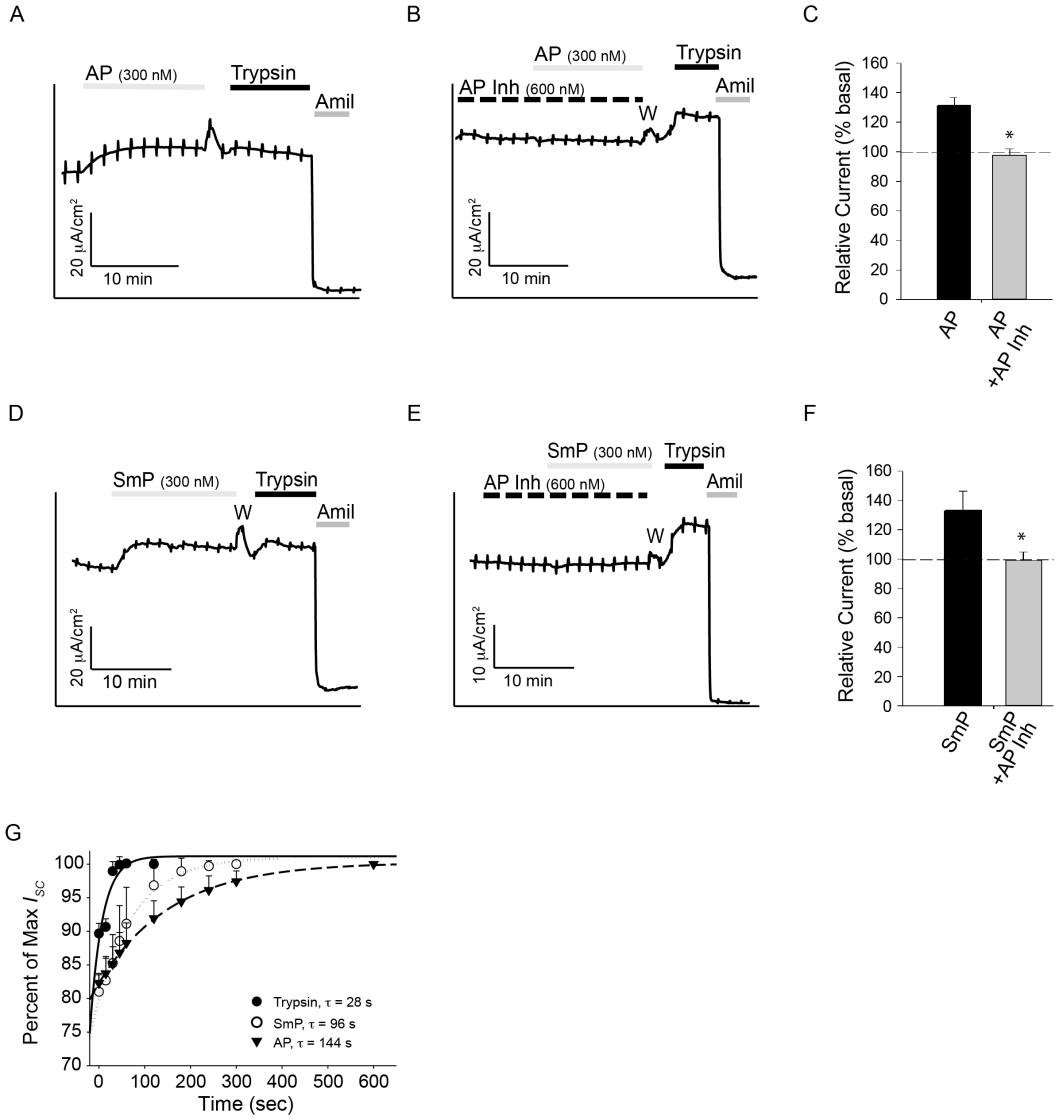
ENaC regulation in mCCD cells by the proteases and the AP inhibitor. The activation of ENaC was assessed using mCCD cells by recording short circuit currents in Ussing chambers. ***A***, a representative trace showing the activation of ENaC in mCCD cells is shown. ***B***, a representative trace of AP treatment after AP Inh pretreatment is shown. ***C***, quantification of the relative currents seen after treatment with either AP or AP and AP Inh for mCCD cells are shown. Basal currents are normalized to 100%. ***D***, a representative trace showing the activation of ENaC by SmP in mCCD cells is shown. ***E***, a representative trace of SmP after AP Inh pretreatment is shown. ***F***, quantification of the relative currents seen after treatment with either SmP or SmP and AP Inh for mCCD cells is shown. ***G***, the kinetics of ENaC activation are shown under conditions of maximal stimulation using trypsin, *closed circles*, AP, *closed triangles*, or SmP, *open circles*. Time constants of ENaC activation are shown for each protease. Serial addition of proteases and inhibitors is shown by the bars above the current trace and labelled. The period in the recording where the apical chamber was washed to remove the serine protease inhibitor, camostat, is indicated with a W. Data shown are representative of at least five experiments. P<0.004, *.

Addition of 300 nM AP resulted in an increase in ENaC activity in the mCCD cells (Figure 4A, C). The kinetics of ENaC activation were slower with AP than with trypsin, as previously reported [26]. This slow activation was sustained and little run down was seen despite prolonged incubation times with AP. To further explore the use of the endogenous AP inhibitor from *Pseudomonas*, the inhibitor was added to the apical bath of Ussing chambers, prior to the addition of protease. The inhibitor alone had no observable impact on baseline ENaC activity (Figure 4B, C). As established *in vitro*, the inhibitor binds tightly to AP and effectively abolishes its activity at near equimolar concentrations (Figure 3). Pretreatment of the apical surface with the 2∶1 ratio of inhibitor: protease blocked the activation of ENaC previously described for AP (Figure 3B, C) in mCCD cells. Treatment with AP Inh effectively blocked the AP-induced ENaC activation seen in the short circuit measurements (p<0.005) (Figure 4C).

To assess the ability of SmP to activate ENaC, the SmP protein was used for short-circuit current recordings. As previously described for AP, addition of SmP to the apical bath of mCCD cells resulted in an increase in ENaC current (Figure 4D). The activation of ENaC was slower and more sustained than that reported for trypsin. The addition of SmP resulted in an increase of ∼35% ENaC activation in mCCD cells, similar to that seen with AP (Figure 4D, F). The AP Inh protein was then used to assess its potential for modulation of the SmP-associated ENaC activation. Pre-incubation with the AP Inh protein resulted in a complete loss of SmP-associated ENaC activation (p<0.005) (Figure 4E, F). This inhibition was seen when the apical chamber was pre-treated with a 2∶1 ratio of inhibitor to protease or when both proteins were added simultaneously. No differences in the inhibition were seen with either treatment regime (data not shown).

Activation of ENaC by both the AP and SmP was kinetically slower than that seen by trypsin under maximal conditions. At maximal dosage, AP activation of ENaC occurred with a time constant ∼5 fold slower than that measured for trypsin (p<0.05) (Figure 4G). Similarly, SmP activation of ENaC occurred with a time constant ∼3.5 fold slower than that of trypsin when evaluated under maximally stimulating conditions (p<0.05). The measured time constants for maximal activation were measured to be 28 seconds for trypsin, 144 seconds for AP and 98 seconds for SmP.

### Functional Regulation of ENaC by AP, SmP and the serralysin inhibitor in HBE cells

**Figure 5:**
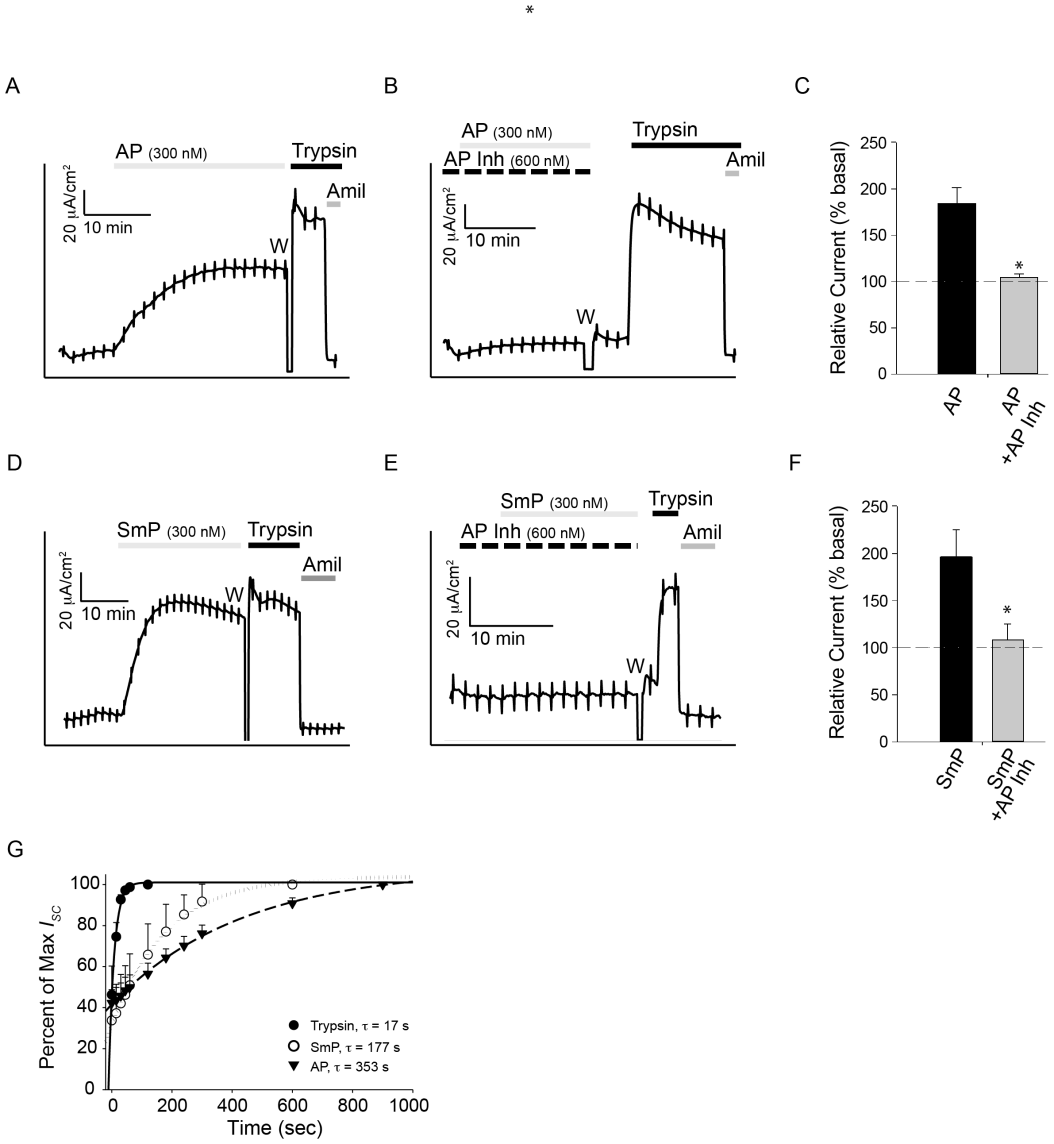
ENaC regulation in HBE cells by the proteases and the AP inhibitor. The activation of ENaC was assessed in HBE cells by recording short circuit currents. ***A***, a representative trace showing the activation of ENaC in HBE cells is shown. ***B***, a representative trace of AP treatment after AP Inh pretreatment is shown. ***C***, quantification of the relative currents seen after treatment with either AP or AP and AP Inh for HBE cells are shown (n = 9). Basal currents are normalized to 100%. ***D***, a representative trace showing the activation of ENaC by SmP in HBE cells is shown. ***E***, a representative trace of SmP after AP Inh pretreatment is shown. ***F***, quantification of the relative currents seen after treatment with either SmP or SmP and AP Inh for HBE cells is shown (n = 5). ***G***, the kinetics of ENaC activation are shown under conditions of maximal stimulation using trypsin, *closed circles*, AP, *closed tringles*, or SmP, *open circles*. Time constants of ENaC activation are shown for each protease. Serial addition of proteases and inhibitors is shown by the bars above the current trace and labelled. The period in the recording where the apical chamber was washed to remove the serine protease inhibitor, camostat, is indicated with a W. Data shown are representative of at least seven experiments. P<0.001, *.

To assess the application of the AP inhibitor in the context of primary airway cells, ENaC activation in human bronchial epithelia (HBE) cell monolayers was assessed. Addition of AP to the apical bath of HBE monolayers resulted in a robust and sustained activation of ENaC (Figure 5A, C). The relative change in HBE ENaC currents was larger than those seen in the mCCD cells, with AP inducing a ∼85% increase in ENaC current. As in the mCCD cells, pretreatment with 2∶1 ratio of inhibitor to protease resulted in a reduction in ENaC activation by the AP protease (Figure 5B, C). The block of ENaC activation was complete with the super-stoichiometric addition of the Inh protein (p<0.001). This inhibition was observed with co-treatment of the AP and Inh proteins or pretreatment with the Inh protein. No measurable differences were observed between the pretreatment or co-treatment application of the inhibitor and protease.

ENaC activation by the SmP protein was similarly assessed in HBE cells. Addition of SmP to the apical chamber resulted in a sustained increase in ENaC activation, similar to that seen in the mCCD cells and with AP (Figure 5D, F). This activation was slow and sustained, resulting in an increase of ∼90% ENaC current in the HBE cells. Treatment with the AP Inh protein blocked this activation when cells were pretreated with the inhibitor or co-treated with the protease and inhibitor (p<0.001) (Figure 5E, F). As with AP and the mCCD cells, no observable changes in ENaC activity were induced by the inhibitor alone. Similarly, both pretreatment with the inhibitor or co-treatment of the inhibitor and protease resulted in a loss of protease-stimulated ENaC activity.

The kinetics of ENaC activation in HBE cells were similarly assessed under conditions of maximal stimulation using the AP and SmP proteins. As with the mCCD cells, the SmP and AP proteins showed different rates of ENaC activation, which were both markedly slower than those observed with trypsin (Figure 5G). Trypsin addition resulted in ENaC activation with a time constant of 17 s, compared to 177 seconds for SmP (p<0.05) and 350 seconds for AP (p<0.05). The changes in HBE ENaC activation kinetics qualitatively paralleled those seen in mCCD cells, though the time constants of activation did differ between the cell lines.

## Discussion

Proteolytic activation of ENaC has been postulated to play a key role in both normal and disease physiologies in the airway [39]–[41]. As such, it is possible that both endogenous (host) and exogenous (pathogen) proteases may play a role in establishing and remodeling the airway environment. Here we demonstrate that multiple members of the serralysin metalloprotease family are capable of activating ENaC. These data suggest that ENaC may serve as a target for the serralysin virulence factors from multiple human pathogens. Further, the *Pseudomonas aeruginosa* AprI, alkaline protease inhibitor can be effectively used to block the *in vitro* activities of purified serralysin proteases and reverse their effects in physiological experiments on cultured and primary epithelial cells.

Our previous studies showed that ENaC can be activated by the addition of AP at the apical surface of cultured and primary epithelial cells [26]. This activation may contribute to the virulence of *Pseudomonas* by remodeling the local airway environment to be more favorable for bacterial adhesion and subsequent colonization. The current study demonstrates that this activation is more general to this class of bacterial exoproteases, as serralysin from *Serratia marcescens* is similarly capable of activating ENaC (Figure 4, 5). This activation is slow when compared to trypsin under maximal stimulating conditions. The slow activation of ENaC by both AP and SmP suggest that the physical basis of activation may also be similar for both proteases. However the kinetics of ENaC activation were slightly accelerated in SmP treated epithelia compared to AP (Figure 4G, 5G), in line with the biophysical characterization of the protease activities (Figure 2).

Binding of the inhibitor to AP and SmP is tight, as measured *in vitro* using purified proteins, and completely abolishes protease activity, consistent with prior reports of binding between the protease and inhibitor (Figure 3) [19]. This tight *in vitro* binding is observed as a complete loss of protease-induced ENaC current in two different model epithelia (Figures 4 and 5). This inhibition provides evidence that the activation of ENaC is mediated through cleavage of a host protein by the bacterial protease. The coincident inhibition of protease activity and loss of ENaC activation suggests that the observed activation is occurring through one or more cleavage events and is not mediated by other non-catalytic binding or protein-protein interactions.

The AP and SmP mediated activation is slow when compared to that elicited by trypsin. The kinetics of ENaC activation by AP and SmP are slowed by ∼3.5 to 20 fold when compared to trypsin in the two cell lines. Though previous studies have demonstrated that cleavage of the γ-subunit is required for AP induced ENaC activation, it is not immediately clear why the activation kinetics vary between the trypsin and the bacterial proteases. The relatively slow and submaximal activation may arise from conformational constraints limiting access to one or more cleavage sites in the ENaC ectodomain. This would be consistent with a model wherein the mechanisms of ENaC cleavage and activation did not co-evolve with the bacterial proteases. Alternatively, this slow activation may be the result of indirect activation via an additional protease-sensitive pathway. Further work to evaluate these differences in activation kinetics is ongoing.

Both the AP and SmP proteases have been implicated in bacterial virulence. Previous studies have suggested that AP is associated with exacerbations in CF and complications in treating *Pseudomonas* [2], [42]–[44]. It is not known if these effects are the result of AP expression, *per se*, or if they are coincident with the expression of AP and other Ca^2+^-regulated virulence factors. While not generally associated with the lung and CF, *Serratia* is often cultured from trachea and is a growing concern as a human pathogen [45], [46]. Adherence and colonization of *Serratia* in the trachea would putatively also be modulated by normal muco-cilliary clearance mechanisms. Alterations in ENaC regulation may disrupt these normal processes and represent one potential mechanism by which serralysin facilitates *Serratia* infection in the trachea.

Consistent with this, recent work evaluating the effects of Liddle syndrome mutants in ENaC demonstrates that tracheal tissue is sensitive to alterations in ENaC activity [47]. Dysregulation of ENaC results in increased Na^+^ flux and an increase in fluid absorption in isolated murine trachea overexpressing β-ENaC under thin film conditions. Similarly, studies of fluid secretion using isolated pig and human trachea and specific channel blockers for CFTR and ENaC demonstrate that both channels contribute to secretion and ASL fluid maintenance [42], [47], [48]. Thus, either inhibition or hyper-activation of these channels would potentially alter fluid balance in the airway [48].

Finally, the inhibition seen with the AP Inh suggests a general mechanism by which this group of protease virulence factors may be partially neutralized. The small, soluble protease inhibitor appears stable and effective for prolonged periods *in vitro* and under a variety of physiological conditions. These characteristics are likely the result of strong selective pressure to protect the pathogen from unregulated intracellular protease activities. Given the strong structural similarity between other members of this family of metalloproteases, it is likely that this inhibitor could inhibit other structurally similar proteases and may be useful in efforts to modulate other serrlaysin or related proteases.
